# Molecular Identification and Phylogenetic Analysis of Laelapidae Mites (Acari: Mesostigmata)

**DOI:** 10.3390/ani13132185

**Published:** 2023-07-03

**Authors:** Evelina Kaminskienė, Jana Radzijevskaja, Loreta Griciuvienė, Michal Stanko, Justina Snegiriovaitė, Dalytė Mardosaitė-Busaitienė, Algimantas Paulauskas

**Affiliations:** 1 Faculty of Natural Sciences, Vytautas Magnus University, Donelaičio Str. 58, LT-44248 Kaunas, Lithuania; evelina.kaminskiene@vdu.lt (E.K.); jana.radzijevskaja@vdu.lt (J.R.); loreta.griciuviene@vdu.lt (L.G.); justina.snegiriovaite@vdu.lt (J.S.); dalyte.mardosaite-busaitiene@vdu.lt (D.M.-B.); 2 Department of Vector-Borne Diseases, Institute of Parasitology, Slovak Academy of Sciences, Hlinkova 3, 04001 Košice, Slovakia; stankom@saske.sk

**Keywords:** Laelapidae mites, 28S ribosomal RNA, cytochrome oxidase subunit I gene, phylogenetic analysis, genetic diversity

## Abstract

**Simple Summary:**

Mites from the family Laelapidae are frequently associated with small mammals, mainly rodents, and can be found on their body surface or in their nests. Classification of the Laelapidae is complicated because of high levels of their morphological and ecological variability. This study aimed to undertake molecular characterization and to assess the phylogenetic relationship among eight Laelapidae mite species collected from different rodent hosts in Lithuania, Norway, Slovakia, and the Czech Republic using the nuclear and mitochondrial molecular markers. Our study provides new molecular data on *Laelaps agilis*, *Laelaps hilaris*, *Laelaps jettmari*, *Haemogamasus nidi*, *Eulaelaps stabularis*, *Hyperlaelaps microti*, *Myonyssus gigas*, and *Hirstionyssus* sp. mites collected from seven different rodent hosts and three geographical regions in Europe. This study, for the first time, registered sequences of four mite species: *H. microti*, *Hirstionyssus* sp., *M. gigas*, and *E. stabularis*.

**Abstract:**

The family Laelapidae (Dermanyssoidea) is morphologically and ecologically the most diverse group of Mesostigmata mites. Although molecular genetic data are widely used in taxonomic identification and phylogenetic analysis, most classifications in Mesostigmata mites are based solely on morphological characteristics. In the present study, eight species of mites from the Laelapidae (Dermanyssoidea) family collected from different species of small rodents in Lithuania, Norway, Slovakia, and the Czech Republic were molecularly characterized using the nuclear (28S ribosomal RNA) and mitochondrial (cytochrome oxidase subunit I gene) markers. Obtained molecular data from 113 specimens of mites were used to discriminate between species and investigate the phylogenetic relationships and genetic diversity among Laelapidae mites from six genera. This study provides new molecular data on *Laelaps agilis*, *Laelaps hilaris*, *Laelaps jettmari*, *Haemogamasus nidi*, *Eulaelaps stabularis*, *Hyperlaelaps microti*, *Myonyssus gigas*, and *Hirstionyssus* sp. mites collected from different rodent hosts and geographical regions in Europe.

## 1. Introduction

Mesostigmata mites represent the most taxon-rich group of Parasitiformes and comprise approximately 11,000 described species [1]. Numerous species of mesostigmatic mites can occasionally infest humans and cause dermatitis and severe allergic reactions. These mites can be potential vectors of the human pathogenic tick-borne encephalitis virus (TBEV) [2] and various rickettsial agents [3,4,5,6]. The superfamily Dermanyssoidea is the largest subdivision of mesostigmatid mites. It consists of 15 families [7], including Laelapidae, which is morphologically and ecologically the most diverse group of Mesostigmata mites [8,9]. Laelapidae currently includes 92 known genera with more than 1300 described species [10,11,12,13]. This family was divided into nine subfamilies: Hypoaspidinae Vitzthum, 1940; Melittiphidinae Evans and Till, 1966; Haemogamasinae Oudemans, 1926; Myonyssinae Bregetova, 1956; Hirstionyssinae Evans and Till, 1960; Mesolaelapinae Tenori and Radovsky, 1974; Alphalaelapinae Tipton, 1960; Laelapinae Berlese, 1892; and Acanthochelinae Radovsky and Gettinger, 1999 [14]. Laelapid mites are frequently associated with small mammals, mainly rodents, and can be found on their body surface or in their nests [15]. Classification of the Laelapidae is complicated. High levels of morphological variability in these mites are causing difficulties. Therefore, molecular evidence is needed to identify mites’ taxonomy at the species level. The phylogenetic analysis provides important information on biodiversity and taxonomy. Most modern taxonomic studies have a total evidence approach incorporating both morphology and DNA sequencing [16,17,18,19,20].

The large (28S) and small (18S) subunit ribosomal RNA (rRNA) genes are most frequently used in taxonomic studies of arthropods [21]. The 18S rRNA gene is generally considered more appropriate for resolving relationships among phyla and superphyla, with the 28S rRNA gene providing more signals at slightly lower taxonomic levels [22,23]. Nuclear rRNA genes have great advantages: they are generally easy to amplify and appear to contain more signals than other genes used for higher-order questions in animal phylogeny [24]. In a previous study, Dowling and OConnor [7] reported the first large-scale phylogenetic relationships within Dermanyssoidea and the evolution of parasitic lineages within the superfamily using the 28S region (domains 1–3) of the nuclear rDNA. With the aim of screening DNA barcodes for mites, in recent studies, Zhao et al. [25] evaluated the universality of the divergent domains with high identification efficiency in Acari. Researchers showed that domains D5, D6, and D8 of 28S rDNA are universal DNA barcodes for molecular classification and identification of mites [25].

The mitochondrial cytochrome c oxidase subunit I (COI) gene was used for the taxonomical identification of mesostigmatic mites and the determination of their intra- and interspecific variation [26,27,28,29]. Recent genetic studies investigated the population genetic structure of *Laelaps agilis* mites across Europe and revealed their phylogenetic relationships [28]. Yang et al. [29] used COI sequence data and morphological characters to assess the phylogenetic relationships of Laelapidae mites from China. However, the phylogenetic relationships and genetic diversity of laelapid mites from Europe are still insufficiently described.

This study aimed to undertake molecular characterization and to assess the phylogenetic relationship among eight Laelapidae mite species collected from different rodent hosts in Lithuania, Norway, Slovakia, and the Czech Republic using the nuclear (28S ribosomal RNA) and mitochondrial (cytochrome oxidase subunit I gene) molecular markers.

## 2. Materials and Methods

### 2.1. Sample Collection

Small rodents were captured with live traps at six locations in Lithuania (Trakai (54°39′24.94′′ N, 24°49′29.48′′ E), Guodžiai peatland (55°58′56.97′′ N, 24°36′50.86′′ E), Curonian Spit (55°33′06.0′′ N, 21°07′31.5′′ E), Rusnė (55°19′26.23′′ N, 21°20′24.15″ E), Beištrakiai (54°54′22.3′′ N, 24°20′28.6′′ E) and Nemunas Loops (54°35′19.04′′ N, 23°59′49.56′′ E)); three locations in Slovakia (Ptičie (48°54′07.3′′ N, 21°55′50.8′′ E), Svetlice (48°34′56.8′′ N, 20°46′37.9′′ E), Hrhov (48°34′53.9′′ N, 20°46′44.4′′ E)); one location in the Czech Republic (České Budějovice (48°59′56.1438′′ N, 14°27′20.217′′ E)); and one location in Norway (Mjävatn (58°32′19.32”N, 8°29′22.92”E)) during 2014–2018.

All trapped rodents were marked and identified by species level and sex. Ectoparasites were collected using soft tweezers, placed into 1.5 mL tubes with 70% ethanol solution, and then stored at 4 °C until processed. The collected mites were determined using morphological identification keys by Mašán, Fend’a [15], Bregetova [30], Baker [31], and Kaminskienė et al. [32].

### 2.2. DNA Extraction

Ammonium hydroxide solution (2.5%) was used for DNA extraction from mites. The laelapid mites were taken from the ethanol solution, dried (3–5 min) on the paper towel at room temperature, and then put in a 0.5 mL microcentrifuge tube. A quantity of 40 µL of 2.5 % NH_4_OH solution was added for each adult mite. In the solution the mites were crushed with a sterile plastic pestle and stored at room temperature for 30 min until incubated at 100 °C for 30 min, allowing for maximal DNA recovery. Subsequently, the tubes were centrifuged at 13,000/min for 1 min to collect condensate from the cap and sides of the tube. All opened tubes with the solution were placed back in the heating block and incubated at 100 °C for 20 min to evaporate the ammonia. After incubation, the tubes were closed and placed on the ice for 2–3 min. Then tubes were centrifuged at 13,000/min for 30 s. Extracted DNA was stored at −20 °C until further usage.

### 2.3. PCR Amplification and Sequencing

Domains 1–3 from the 28S nuclear ribosomal RNA gene region and the COI gene of mitochondrial DNA were used for molecular characterization and phylogenetic reconstruction within the family Laelapidae [33].

Conventional PCR was performed to amplify 856 bp fragment of mites 28S rRNA using 43F 5′- GCT GCG AGT GAA CTG GAA TCA AGC CT3′ and 929R 5′-AGG TCA CCA TCT TTC GGG TC-3′ primers [7]. Each 25 µL reaction contained a mixture of 13.7 µL ddH_2_O, 2.5 µL 10× PCR buffer (KCl-(NH_4_) SO_4_) (Thermo Fisher Scientific Baltics, Vilnius, Lithuania), 20 µL 25 mM MgCl_2_, 0.5 µL 25 mM dNTP, 1 µL of each 10 mM primer, 0.3 µL Taq DNA Polymerase (Thermo Fisher Scientific Baltics, Vilnius, Lithuania) (5 U/μL), and 4 µL DNA. The PCR reaction conditions were as follows: initial denaturation at 94° for 2 min; followed by 35 cycles of denaturation at 94° for 25 s, annealing at 53° for 20 s, and extension at 72° for 1min; with a final extension at 72° for 7 min after completion of all cycles.

To amplify a 709 bp fragment of the COI gene, conventional PCR with primers LCO1490 (5’-GGT CAA CAA ATC ATA AAG ATA TTG G-3’) and HCO2198 (5’-TAA ACT TCA GGG TGA CCA AAA AAT CA-3’) was performed [33]. Each 25 µL reaction contained a mixture of 16.5 µL ddH_2_O, 5 µL 5× MyTaq reaction buffer (Thermo Fisher Scientific Baltics, Vilnius, Lithuania), 0.5 µL of each 10 mM primer, 0.5 µL MyTaq DNA polymerase (Thermo Fisher Scientific Baltics, Vilnius, Lithuania) (5 U/μL), and 2 µL DNA. The PCR reaction conditions were as follows: initial denaturation at 94° for 3 min; followed by 40 cycles of denaturation at 94° for 45 s, annealing at 45° for 45 s, and extension at 72° for 1 min; with a final extension at 72° for 5 min after completion of all cycles.

PCR products were subjected to electrophoresis on 1.5% agarose gel and analyzed by UV transilluminator. The DNA fragment was excised from agarose gel and purified using a GenJET PCR purification kit (Thermo Fisher Scientific Baltics, Vilnius, Lithuania) according to the manufacturer’s protocol. All purified PCR products were sent for DNA sequencing to a sequencing service (Macrogen, Amsterdam, The Netherlands).

### 2.4. Sequence Analysis

The sequences obtained in this study were analyzed using the BLAST program to confirm the morphological identification of mite species and were aligned with the corresponding sequences of other laelapid mites available in GenBank using ClustalW [34] multiple alignments implemented in MegaX [35]. The partial 28S rRNA and COI gene sequences were aligned in two independent datasets. The intraspecific and interspecific pairwise genetic distances, variable sites, conserved sites, and parsimony-informative sites were computed by Mega X. The non-synonymous mutation rate (Ka) and synonymous mutation rate (Ks), haplotype diversity (Hd), nucleotide diversity (Π), and polymorphic sites (S) were calculated using DnaSP v5.10.01 [36]. The representative sequences of 28S rRNA and COI gene were deposited to GenBank. 

### 2.5. Phylogenetic Analysis

Phylogenetic trees were constructed using maximum likelihood (ML) and Bayesian inference (BI) methods. The best-fitting nucleotide substitution model (GTR + I + G) was determined by the Bayesian Information Criterion (BIC) yielded using jModelTest v2.1.10 [37]. The ML trees were generated using the Tamura–Nei parameter model in MEGA X, with each node supported by 1000 bootstraps. Bayesian inference (BI) analyses were run with MrBayes v.3.2.7 [38]. The Markov chain was run with 40,000,000 generations, and trees were sampled every 1000th generation. The first 25% of samples were discarded as burn-in, and the remaining saved samples were used to estimate the posterior probabilities (PP) of each bipartition. The phylogenetic tree was visualized using FigTree v1.4.4 [39].

To estimate the phylogenetic relationships among the COI gene haplotypes of *L. agilis* derived from different rodent hosts and geographical regions, median-joining (MJ) networks were constructed using Network 10.2.0.0 [40].

## 3. Results

### 3.1. 28S rRNA Region

A total of 53 sequences of partial 28S rRNA gene were obtained from eight species of Laelapidae mites (*Laelaps agilis*, *Laelaps jettmari*, *Laelaps hilaris*, *Haemogamasus nidi*, *Eulaelaps stabularis*, *Hyperlaelaps microti*, *Myonyssus gigas*, and *Hirstionyssus* sp.) collected from seven small rodent species (*Apodemus flavicollis*, *Apodemus agrarius*, *Apodemus sylvaticus*, *Clethrionomys glareolus*, *Microtus arvalis*, *Micromys minutus*, and *Microtus oeconomus*) in Lithuania, Slovakia, the Czech Republic, and Norway (Table 1). The lengths of the analyzed 28S rRNA sequences varied between 527 and 821 bp; the AT content ranged from 55.5 to 57.2% (Table 1). Sequence comparison showed 174 variable sites among 28S rRNA gene sequences of all examined Laelapidae mites and 51 variable sites among mites from the *Laelaps* genus (Table 2).

Partial 28S rRNA sequences obtained from *L. agilis* (MZ043837–MZ043844), *L. hilaris* (MZ043845, MZ043846), *L. jettmari* (MZ043833, MZ043834, ON763742), *M. gigas* (MZ043831, MZ043832), and *Hirstionyssus* sp. (ON775520, ON775521) showed no intraspecific variability (Table 1). However, two genotypes of *H. microti* (MZ043835; MZ043836), *E. stabularis* (MZ043828, MZ043829, MZ043830), and *Hg. nidi* (MZ061928, MZ061929, MZ061930, MZ061931) were identified. *H. microti* sequences differed at one nucleotide position showing ambiguous nucleotide Y (C/T—transition). Two genotypes of *E. stabularis* detected in Lithuania (MZ043828; *n* = 2) and Norway (MZ043829; MZ043830) were specific to their respective locations (Table 1) and differed at two nucleotide positions. Two genotypes representing six 28S rRNA sequences derived from *Hg. nidi* differed at three nucleotide positions (three sequences (MZ061928-MZ061930) had one ambiguous nucleotide W (A/T transversion).

The overall mean genetic distance between laelapid mite sequences obtained in this study was 0.0820. The intra- and interspecific genetic distances of Laelapidae species are shown in Table 3. The highest interspecific distances were detected between *H. microti* and the other Laelapidae mite species.

The phylogenetic analysis based on 28S rRNA gene included sequences of other dermanysoid mite species available in GenBank: *L. jettmari* (*pavlovskyi*) (GU440635), *L. hilaris* (GU440637), *Laelaps stupkai* (GU440596), *Laelaps clethrionomydis* (GU440636), *Laelaps kochi* (GU440626), *Laelaps muris* (GU440638), *Ondatralaelaps multispinosus* (FJ911778), *Laelaps vansomereni* (GU440619), *Laelaps zumpti* (GU440623), *Laelaps spinigera* (GU440613), *Laelaps mazzai* (GU440590), *Haemogamasus reidi* (GU440583), *Brevisterna morlani* (FJ911773), *Haemogamasus* sp. (FJ911772), and *Dermanyssus gallinae* (FJ911771).

**Figure 1:**
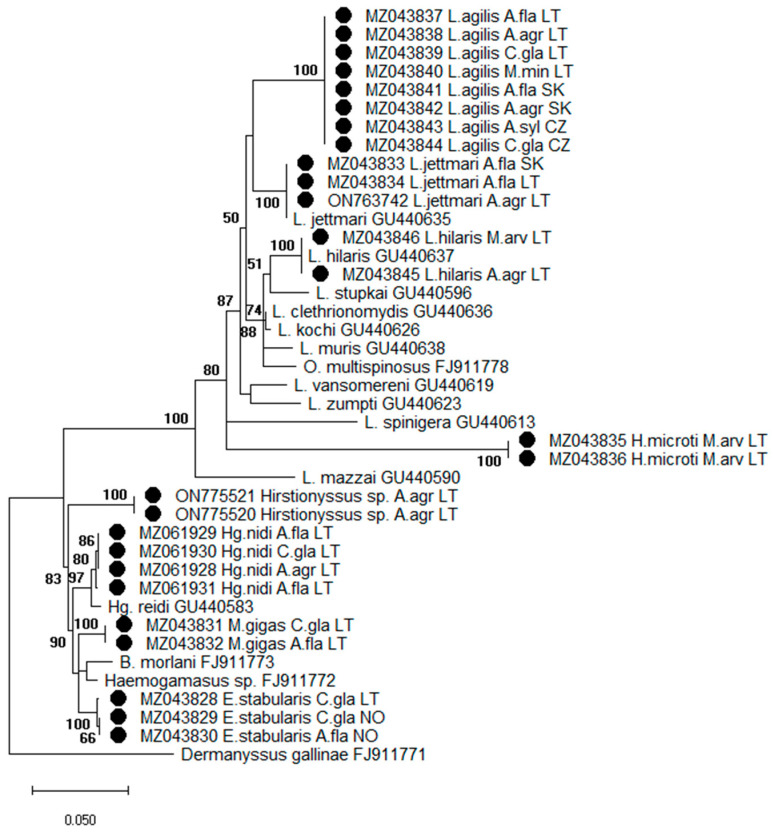
Phylogenetic tree of *28S rRNA* gene sequences of Laelapidae mites generated using the maximum likelihood method and Tamura–Nei model and bootstrap analysis of 1000 replicates. Samples sequenced in the present study are marked (●). Abbreviations: A. agr—*Apodemus agrarius*, A. fla—*Apodemus flavicollis*, A. syl—*Apodemus sylvaticus*, M. arv—*Microtus arvalis*, C. gla—*Clethrionomys glareolus*, M. min—*Micromys minutus*, LT—Lithuania, SK—Slovakia, CZ—Czech Republic, NO—Norway.

The phylogenetic tree of 28S rRNA gene sequences constructed using the ML method is divided into two main clusters: one cluster groups sequences of twelve *Laelaps* genus species and *H. microti*, while the other cluster consists of six species of *Hirstionyssus, Haemogamasus, Myonyssus, Brevisterna,* and *Eulaelaps* genera. The members of each species form individual subclusters on the phylogenetic tree (Figure 1).

The 28S rRNA gene sequences of *L. jettmari* and *L. hilaris* obtained in the present study were 100% identical to corresponding sequences derived from GenBank: GU440635 and GU440637, respectively (Figure 1). Sequences of *Hg. nidi* (MZ061928, MZ061929, MZ061931, MZ061930) collected in Lithuania shared 98.95–99.08% similarity to *Hg. reidi* (synonym *Hg. nidi*) sequences from GenBank: GU440583.

### 3.2. COI Gene

The partial sequences of the COI gene were successfully obtained from six species of Laelapidae mites (*L. agilis*, *L. jettmari*, *L. hilaris*, *Hg. nidi*, *H. microti*, and *M. gigas*) collected from six species of small rodents (*A. flavicollis*, *A. agrarius*, *A. sylvaticus*, *C. glareolus*, *M. arvalis* and *M. oeconomus*). A total of 60 good-quality COI sequences were analyzed (among them 47 sequences of *L. agilis*, four sequences of *L. jettmari*, three sequences of *L. hilaris*, two sequences of *Hg. nidi*, two sequences of *M. gigas*, and two sequences of *H. microti*). COI sequences of Laelapidae mites ranged from 582 to 699 bp in length and from 64.9 to 74.6% in AT content (Table 4); there were 253 variable sites, 330 conserved sites, and 245 parsimony-informative sites. A total of 23 nucleotide variable sites were detected among *L. agilis* species (Table 5). The mean value of Ka/Ks of COI gene sequences obtained in this study was 2.31.

**Figure 2:**
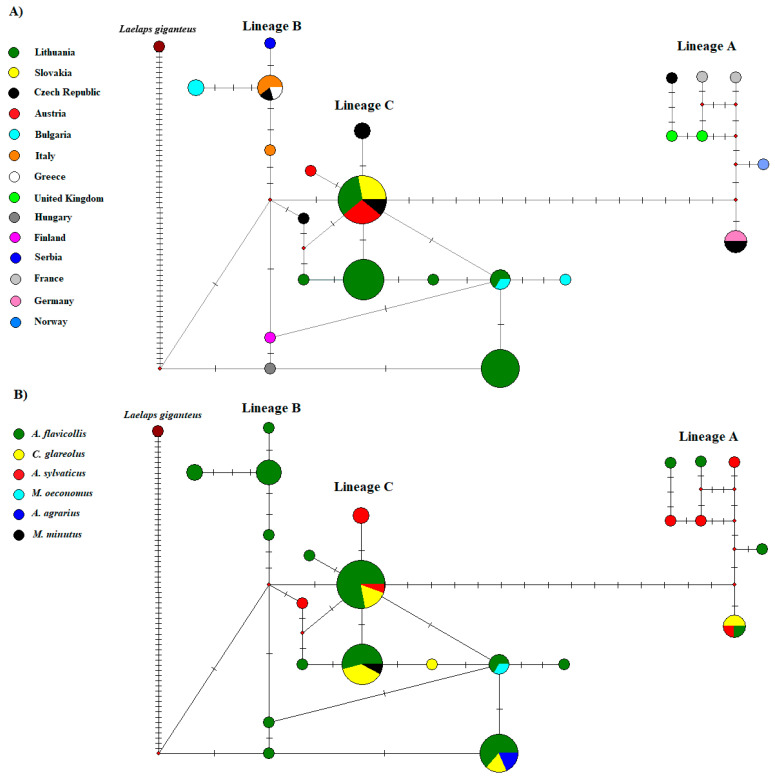
A median-joining network of haplotypes based on COI sequences of *L. agilis* from different European regions. The circles represent different haplotypes with size proportional to relative frequencies. (**A**): different colors represent geographic distribution; (**B**): different colors represent host species. The network branches linking the cycles indicate one mutation step; two or more mutations are represented by slashes crossed with the network branches. The red points indicate undetected intermediate haplotypes.

Nine COI haplotypes (h = 9) between 23 *L. agilis* sequences were detected with estimated haplotype diversity of Hd = 0.870, nucleotide diversity Π = 0.00720, and a total number of polymorphic sites S = 23. In total, 559 conserved sites, one singleton site, and 19 parsimony-informative sites were detected. Haplotype H_1 of *L. agilis* was the most frequent. It was found in three out of four different locations (Lithuania, Slovakia, and the Czech Republic) (Table 4, Figure 2). Haplotypes H_2 and H_3 (the Czech Republic), H_4-H_8 (Lithuania), and H_9 (Norway) of *L. agilis* were specific for their respective sampling locations.

**Figure 3:**
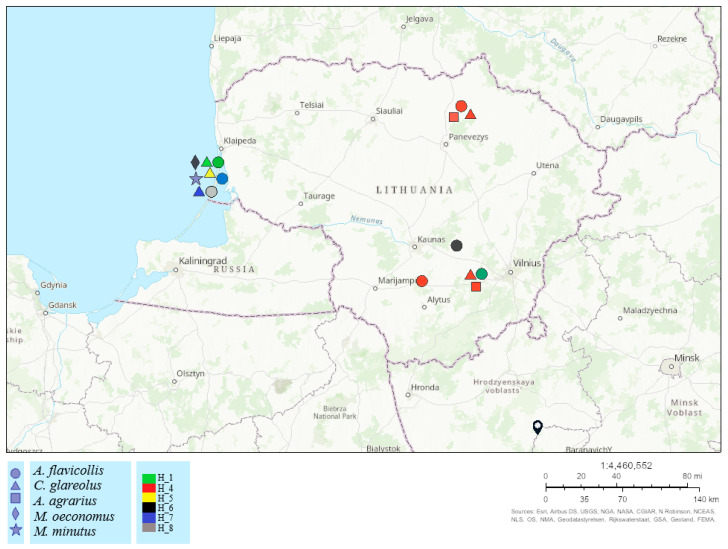
The distribution of COI haplotypes of *L. agilis* collected from different host species and sampling sites in Lithuania. Different host species and haplotypes are shown by various shapes and colors. The number of samples varied from 1 to 7.

In this study, six haplotypes of *L. agilis* were detected in Lithuania. From these sequences, four haplotypes of *L. agilis* detected in Lithuania (H_4, H_5, H_7, and H_8) were unique and differed from the most similar sequences in GenBank (Figure 2A). The distribution of *L. agilis* haplotypes in different areas of Lithuania showed that the highest haplotype diversity was detected in the Lithuanian coastal area—the Curonian Spit where five of six haplotypes (H_1, H_5-H_8; *n* = 21) were found. In the continental part of the country (northern and south-eastern parts), three haplotypes were detected (H_1, H_4, H_6; *n* = 13) (Figure 3). Distribution of different *L. agilis* haplotypes did not reveal specificity to host species. Five haplotypes were detected in *A. flavicollis*, four haplotypes in *C. glareolus*, and *A. agrarius*, *M. oeconomus*, and *M. minutus* each harbored one haplotype H_4, H_6, and H_7, respectively. This study detected three COI haplotypes of *L. jettmari* (*n* = 4) and two COI haplotypes of *H. microti* (*n* = 2). In contrast, only one haplotype was found among *L. hilaris*, *Hg. Nidi*, and *M. gigas* sequences (Table 4).

The overall mean genetic distance between laelapid mites’ COI gene sequences obtained in this study was 0.1215. The inter- and intraspecific genetic distances based on the COI gene are shown in Table 3. The highest interspecific distances were detected between *M. gigas* and the other Laelapidae mite species. The intraspecific genetic distance among *L. agilis* sequences was 0.0074.

**Figure 4:**
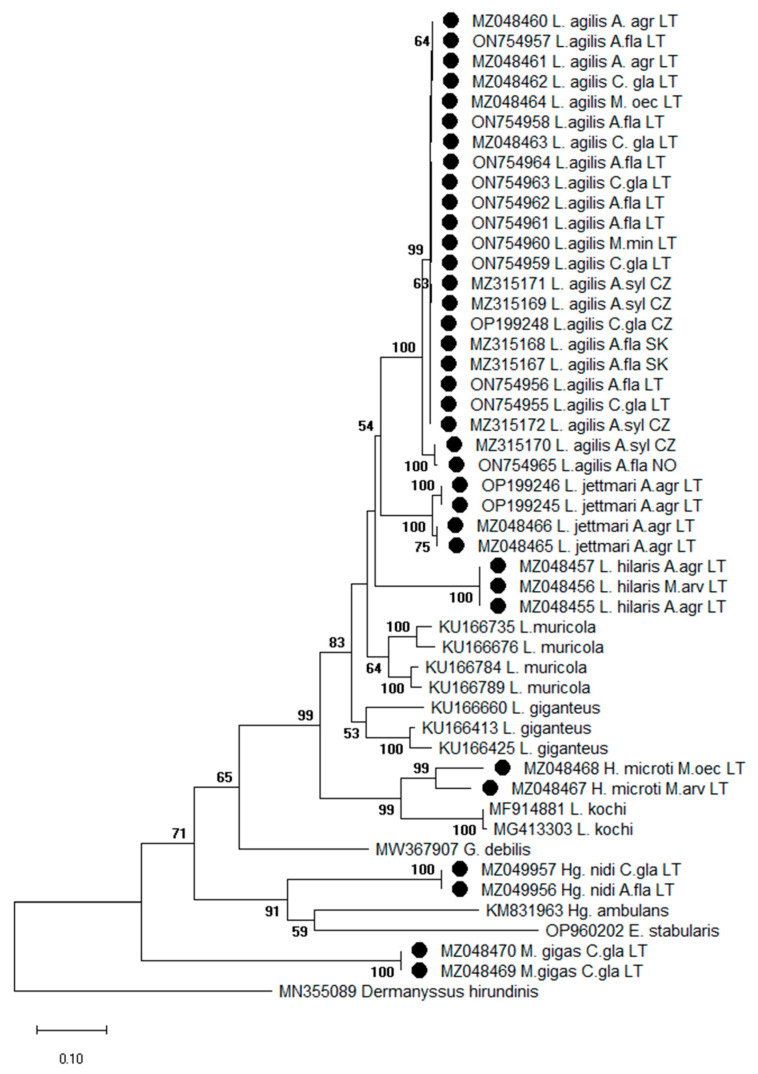
Phylogenetic tree of COI gene sequences of Laelapidae mites generated using the maximum likelihood method and General Time Reversible model (Gamma Distributed with Invariant Sites (G + I)) model and bootstrap analysis of 1000 replicates. Samples sequenced in the present study are marked (●). Abbreviations: *A. agr– Apodemus agrarius*, *A. fla—Apodemus flavicollis*, *A. syl—Apodemus sylvaticus*, *M. arv—Microtus arvalis*, *M. oec—Microtus oeconomus*, *C. gla—Clethrionomys glareolus*, *M. min—Micromys minutus*, LT—Lithuania, SK—Slovakia, CZ—Czech Republic, NO—Norway.

The phylogenetic analysis based on the COI gene included sequences of other dermanysoid mite species available in GenBank: *Laelaps muricola* (KU166735; KU166676; KU166784; KU166789), *Laelaps giganteus* (KU166660; KU166413; KU166425), *L. kochi* (MF914881; MG413303), *Haemogamasus ambulans* (KM831963), *Gaeolaelaps debilis* (MW367907), *E. stabularis* (OP960202), and *Dermanyssus hirundinis* (MN355089). The phylogenetic tree of COI gene sequences constructed using the ML method showed a clear separation of different species of Laelapidae mites into different clusters. *L. agilis* sequences were heterogenic and, together with *L. jettmari* and *L. hilaris*, formed a separate cluster on the phylogenetic tree (Figure 4).

**Figure 5:**
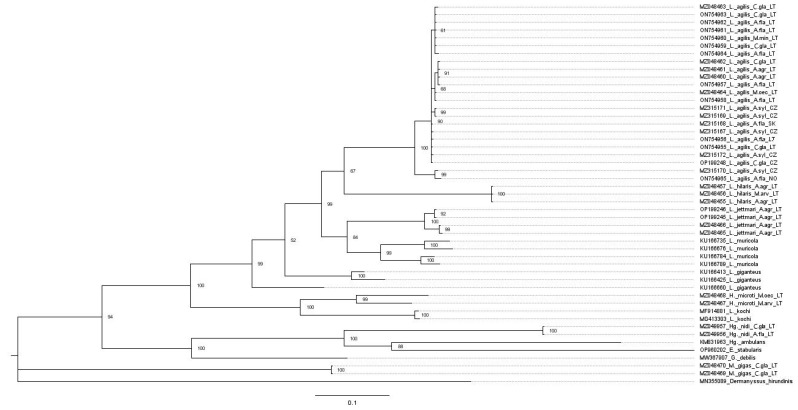
A Bayesian phylogenetic tree of the Laelapidae mites, based on the COI gene sequences. Numbers above the branches show the Bayesian posterior probabilities. Samples sequenced in the present study are marked (●). Abbreviations: *A. agr—Apodemus agrarius*, *A. fla—Apodemus flavicollis*, *A. syl—Apodemus sylvaticus*, *M. arv—Microtus arvalis*, *M. oec—Microtus oeconomus*, *C. gla—Clethrionomys glareolus*, *M. min—Micromys minutus*, LT—Lithuania, SK—Slovakia, CZ—the Czech Republic, NO—Norway. The geographical structure of *L. agilis* was supported by the median-joining network, which showed at least three major Lineages A, B, and C (Figure 2A,B). In the median-joining (MJ) network were included the COI gene haplotypes of *L. agilis* (derived from different rodent hosts and geographical regions) detected in our study and available in GenBank. Haplotypes assigned to Lineage A were found in Norway, Finland, the United Kingdom, France, and the Czech Republic. Most of the haplotypes were not shared between different geographic areas, except for one haplotype identified in Germany and the Czech Republic (Figure 2A). This lineage (A) showed haplotype sharing between three host species: *A. flavicollis*, *A. sylvaticus*, *C. glareolus* (Figure 2B). A single haplotype was assigned to Lineage B, shared between the *L. agilis* from Italy, Greece, and the Czech Republic, and three unique to the individuals from Italy, Bulgaria, and Serbia. This lineage (B) only included mites collected from *A. flavicollis*. All sequences of *L. agilis* from Lithuania belonged to Lineage C. They clustered together with samples from Slovakia, Bulgaria, Austria, the Czech Republic, Hungary, and Finland. Additionally, the network showed that four haplotypes have so far been found only in Lithuania (Figure 2A). This lineage (C) was composed of *L. agilis* found in different host species (*A. flavicollis*, *A. sylvaticus*, *A. agrarius*, *C. glareolus*, *M. minutus*, and *M. oeconomus*) (Figure 2B and Figure 3).

Another phylogenetic tree of Laelapidae mites was constructed using the BI method (Figure 3). ML and BI phylogenetic trees differed slightly in topology and branching structures (Figure 4 and Figure 5). The Bayesian tree (Figure 5) exhibited higher posterior probabilities (PPs) values (52–100%) than the bootstrap values (38–100%) of the ML (Figure 4).

## 4. Discussion

In the present study, eight species of Laelapidae mites collected from different rodent hosts and geographical regions in Europe were molecularly characterized based on both nuclear 28S rRNA and mitochondrial COI gene regions. Our findings confirm that these molecular markers could be successfully used for molecular identification of Laelapidae mite species and inference of their phylogenetic relationships [7,27,28,29]. On the other hand, mitochondrial DNA evolves much faster and is more evolutionarily variable than the ribosomal DNA of the nuclear genome [41]. Thus, the COI gene sequences are more appropriate for analyzing intraspecific phylogenetic relationships [26,42]. In this study, our results based on the COI gene indicated a high intraspecific variation (9 haplotypes out of 23 obtained sequences) in *L. agilis* species. Intraspecific variations on the COI gene were also detected in *L. jettmari* (three haplotypes identified among four obtained sequences) and *H. microti* (two haplotypes among two obtained sequences).

Our findings provide new data on the intra- and interspecific phylogenetic relationships of Laelapidae mites belonging to six genera. This study, for the first time, registered sequences of four mite species: *H. microti*, *Hirstionyssus* sp., *M. gigas*, and *E. stabularis*.

Phylogenetic relationships based on 28S rRNA exhibited polyphyly of the different species from the family Laelapidae. The previous study also determined a polytomy structure in the phylogenetic relationships [7]. In contrast, Li et al. [43] and Yang et al. [44] showed that based on mitochondrial barcoding region, the family Laelapidae is a monophyletic group.

The results of the phylogenetic analysis based on 28S rRNA revealed the separation of Laelapidae mites into two different groups. The first group consists of sequences belonging to obligate parasitic mites from two genera, *Laelaps* and *Hyperlaelaps*. The second group contains two clusters—one cluster consists of sequences belonging to facultative parasitic mites *Eulaelaps*, *Haemogamasus*, and *Myonyssus*, whereas sequences of obligate parasitic *Hirstionyssus* sp. formed a separate cluster (Figure 1).

It should be noticed that phylogenetic analysis based on both genes (28S rRNA and COI) indicated the clustering of *H. microti* with the species of the genus *Laelaps* and did not show separation into distinct clades. The differences between the molecular and morphological taxonomy of this species were also observed in recent studies [29,44].

In line with a previous study [28], our results of the phylogenetic analysis based on mt DNA also corroborated three lineages (Lineages A, B, and C) within *L. agilis* (Figure 2). The results did not indicate clear specificity according to geographical locations. Lineages A and C comprised specimens from diverse geographical regions of Europe (North, Central-Eastern, and West) (Figure 2A), which was also revealed in a recent study [28]. However, our results supplemented Lineage A with one specimen from Norway and Lineage C with sequences from Lithuania (Figure 2A). Moreover, our findings showed no clear host species specificity and confirmed the results previously obtained by Nazarizadeh et al. [28]. However, the number of host species in these lineages (A and C) was supplemented by three additional species (*A. agrarius*, *M. minutus*, and *M. oeconomus*) in this study. Only one *L. agilis* lineage (B) showed clear specificity according to host species (*A. flavicollis*) (Figure 2B), and it is consistent with the results of the Nazarizadeh et al. study [28].

Considering several species of rodents as important hosts of the parasitic mites analyzed in this study, it should be mentioned that populations of rodents of the genera *Apodemus* and *Clethrionomys* in Europe are genetically heterogeneous. During the glaciation in the Quaternary, they survived in various refugia in southern Europe [45,46] and had complex recolonization routes in Europe. A specific species in this regard is *Apodemus agrarius*, which only relatively recently colonized Europe from Asia [47].

Based on published data, at least 21 parasitic mite species belonging to the Laelapidae family have been morphologically identified in Lithuania [32,48,49,50,51]. This study provides the first molecular characterization of eight species of laelapid mites collected from different rodent hosts in Lithuania. Therefore, the more comprehensive phylogenetic analysis of Laelapidae mites in Lithuania must be further investigated.

## 5. Conclusions

Our study provides new molecular data on *Laelaps agilis*, *Laelaps hilaris*, *Laelaps jettmari*, *Haemogamasus nidi*, *Eulaelaps stabularis*, *Hyperlaelaps microti*, *Myonyssus gigas*, and *Hirstionyssus* sp. mites collected from seven different rodent hosts and three geographical regions in Europe. This study is the first molecular characterization of eight Laelapidae mite species in Baltic countries. Specifically, 28S rRNA and COI sequences of four mite species were, for the first time, registered in the NCBI database (2021–2022).

## Figures and Tables

**Table 1:** Hosts and GenBank nucleotide accession numbers of the 28S rRNA gene region sequences of Laelapidae mites.

Taxonomic Status of Species	Host Species	Country	Length (bp)	GenBank Accession No.	AT%	No of Representative Samples
Genus *Laelaps*						
*L. agilis*	*A. agr*	Lithuania	527	MZ043838	56.4	3
	*A. fla*	Lithuania	760	MZ043837	56.8	3
	*C. gla*	Lithuania	527	MZ043839	56.4	1
	*M. min*	Lithuania	818	MZ043840	56.6	1
	*A. agr*	Slovakia	818	MZ043842	56.7	1
	*A. fla*	Slovakia	805	MZ043841	56.8	8
	*A. syl*	Czech Republic	818	MZ043843	56.7	8
	*C. gla*	Czech Republic	805	MZ043844	56.8	1
*L. jettmari*	*A. fla*	Lithuania	802	MZ043834	57.2	1
	*A. fla*	Slovakia	803	MZ043833	57.2	4
	*A. agr*	Lithuania	821	ON763742	57.1	1
*L. hilaris*	*A. agr*	Lithuania	814	MZ043845	56.6	1
	*M. arv*	Lithuania	805	MZ043846	56.8	1
Genus *Eulaelaps*						
*E. stabularis* (1 gen.)	*C. gla*	Lithuania	820	MZ043828	57.0	2
*E. stabularis* (2 gen.)	*A. fla*	Norway	743	MZ043830	55.9	1
	*C. gla*	Norway	770	MZ043829	56.1	1
Genus *Haemogamasus*						
*Hg. nidi* (1 gen.)	*A. agr*	Lithuania	768	MZ061928	55.9	1
	*A. fla*	Lithuania	768	MZ061929	55.9	1
	*C. gla*	Lithuania	768	MZ061930	55.9	1
*Hg. nidi* (2 gen.)	*A. fla*	Lithuania	768	MZ061931	55.8	3
Genus *Hyperlaelaps*						
*H. microti* (1 gen.)	*M. arv*	Lithuania	760	MZ043835	55.8	1
*H. microti* (2 gen.)	*M. arv*	Lithuania	760	MZ043836	55.9	1
Genus *Myonyssus*						
*M. gigas*	*A. fla*	Lithuania	771	MZ043832	55.9	3
	*C. gla*	Lithuania	811	MZ043831	55.5	2
Genus *Hirstionyssus*						
*Hirstionyssus* sp.	*A. agr*	Lithuania	818	ON775520	55.9	1
	*A. agr*	Lithuania	818	ON775521	55.9	1

Abbreviations: *A. agr—Apodemus agrarius*, *A. fla—Apodemus flavicollis*, *A. syl—Apodemus sylvaticus*, *M. arv—Microtus arvalis*, *C. gla—Clethrionomys glareolus*, *M. min—Micromys minutus*.

**Table 2:** Comparison of the 28S rRNA gene sequences of *Laelaps* genus mites in this study.

Nucleotide Position							1	1	1	2	2	2	3	3	3	3	3	3	3	3	3	3	3	3	3	4
	1	2	3	8	8	9	0	0	1	8	9	9	1	3	4	6	6	6	7	7	7	8	8	8	9	0
Samples	7	8	3	3	6	0	7	9	5	9	5	9	7	5	8	3	6	7	3	4	9	1	3	5	6	5
MZ043837 *L. agilis A. flavicollis* Lithuania	G	G	T	A	T	G	A	G	T	G	T	G	C	T	A	G	T	G	A	A	G	C	G	T	T	C
MZ043838 *L. agilis A. agrarius* Lithuania	.	.	.	.	.	.	.	.	.	.	.	.	.	.	.	.	.	.	.	.	.	.	.	.	.	.
MZ043839 *L. agilis C. glareolus* Lithuania	.	.	.	.	.	.	.	.	.	.	.	.	.	.	.	.	.	.	.	.	.	.	.	.	.	.
MZ043840 *L. agilis M. minutus* Lithuania	.	.	.	.	.	.	.	.	.	.	.	.	.	.	.	.	.	.	.	.	.	.	.	.	.	.
MZ043841 *L. agilis A. flavicollis* Slovakia	.	.	.	.	.	.	.	.	.	.	.	.	.	.	.	.	.	.	.	.	.	.	.	.	.	.
MZ043842 *L. agilis A. agrarius* Slovakia	.	.	.	.	.	.	.	.	.	.	.	.	.	.	.	.	.	.	.	.	.	.	.	.	.	.
MZ043843 *L. agilis A. sylvaticus* Czech Republic	.	.	.	.	.	.	.	.	.	.	.	.	.	.	.	.	.	.	.	.	.	.	.	.	.	.
MZ043844 *L. agilis C. glareolus* Czech Republic	.	.	.	.	.	.	.	.	.	.	.	.	.	.	.	.	.	.	.	.	.	.	.	.	.	.
MZ043846 *L. hilaris M. arvalis* Lithuania	C	A	.	T	C	A	G	A	A	T	C	A	T	C	T	T	C	.	G	G	.	.	A	G	A	A
MZ043833 *L. jettmari A. flavicollis* Slovakia	T	.	A	T	.	.	.	A	G	.	.	.	T	.	T	.	C	A	G	G	A	T	.	G	A	A
MZ043834 *L. jettmari A. flavicollis* Lithuania	T	.	A	T	.	.	.	A	G	.	.	.	T	.	T	.	C	A	G	G	A	T	.	G	A	A
ON763742 *L. jettmari A. agrarius* Lithuania	T	.	A	T	.	.	.	A	G	.	.	.	T	.	T	.	C	A	G	G	A	T	.	G	A	A
**Nucleotide Position**		**4**	**4**	**4**	**4**	**4**	**4**	**4**	**4**	**4**	**4**	**4**	**4**	**5**	**5**	**5**	**5**	**5**	**5**	**5**	**5**	**5**	**5**	**5**	**6**	**7**
		**0**	**1**	**1**	**2**	**4**	**5**	**7**	**7**	**8**	**8**	**9**	**9**	**1**	**1**	**1**	**2**	**4**	**7**	**8**	**8**	**9**	**9**	**9**	**0**	**2**
**Samples**		**9**	**0**	**8**	**9**	**5**	**9**	**4**	**6**	**4**	**7**	**2**	**5**	**2**	**3**	**4**	**6**	**4**	**4**	**4**	**8**	**0**	**6**	**7**	**7**	**9**
MZ043837 *L. agilis A. flavicollis* Lithuania		G	G	A	A	G	T	A	A	C	C	T	T	G	A	G	T	A	T	A	T	T	A	C	C	C
MZ043838 *L. agilis A. agrarius* Lithuania		.	.	.	.	.	.	.	.	.	.	.	.	.	.	.	.	.	.	.	.	.	.	.	.	.
MZ043839 *L. agilis C. glareolus* Lithuania		.	.	.	.	.	.	.	.	.	.	.	.	.	.	.	.	.	.	.	.	.	.	.	.	.
MZ043840 *L. agilis M. minutus* Lithuania		.	.	.	.	.	.	.	.	.	.	.	.	.	.	.	.	.	.	.	.	.	.	.	.	.
MZ043841 *L. agilis A. flavicollis* Slovakia		.	.	.	.	.	.	.	.	.	.	.	.	.	.	.	.	.	.	.	.	.	.	.	.	.
MZ043842 *L. agilis A. agrarius* Slovakia		.	.	.	.	.	.	.	.	.	.	.	.	.	.	.	.	.	.	.	.	.	.	.	.	.
MZ043843 *L. agilis A. sylvaticus* Czech Republic		.	.	.	.	.	.	.	.	.	.	.	.	.	.	.	.	.	.	.	.	.	.	.	.	.
MZ043844 *L. agilis C. glareolus* Czech Republic		.	.	.	.	.	.	.	.	.	.	.	.	.	.	.	.	.	.	.	.	.	.	.	.	.
MZ043846 *L. hilaris M. arvalis* Lithuania		.	A	G	G	T	C	T	G	.	G	C	A	T	T	.	C	T	.	G	A	.	T	.	T	T
MZ043833 *L. jettmari A. flavicollis* Slovakia		A	A	.	G	A	.	T	G	T	G	C	.	.	.	A	C	C	C	.	.	C	T	T	T	T
MZ043834 *L. jettmari A. flavicollis* Lithuania		A	A	.	G	A	.	T	G	T	G	C	.	.	.	A	C	C	C	.	.	C	T	T	T	T
ON763742 *L. jettmari A. agrarius* Lithuania		A	A	.	G	A	.	T	G	T	G	C	.	.	.	A	C	C	C	.	.	C	T	T	T	T

**Table 3:** Genetic distances within and between Laelapidae species.

Species	Genetic Distance			
	28S rRNA		COI	
	Within Species	Between Species ^a^	Within Species	Between Species ^a^
*L. a*	0	0–0.046312 (vs. *L. j*)	0–0.033718	0–0.125923 (vs. *L. j*)
		0–0.056138 (vs. *L. h*)		0–0.146954 (vs. *L. h*)
		0–0.116236 (vs. *E. s*)		- (vs. *E. s*)
		0–0.113036 (vs. *Hg. n*)		0–0.302688 (vs. *Hg. n*)
		0–0.137949 (vs. *H. m*)		0–0.211204 (vs. *H. m*)
		0–0.113032 (vs. *M. g*)		0–0.308024 (vs. *M. g*)
		0–0.112793 (vs. *Hirst.* sp.)		- (vs. *Hirst.* sp.)
*L. j*	0	0–0.042192 (vs. *L. h*)	0–0.019213	0–0.155239 (vs. *L. h*)
		0–0.099372 (vs. *E. s*)		- (vs. *E. s*)
		0–0.100931 (vs. *Hg. n*)		0–0.318748 (vs. *Hg. n*)
		0–0.125555 (vs. *H. m*)		0–0.206667 (vs. *H. m*)
		0–0.099202 (vs. *M. g*)		0–0.337406 (vs. *M. g*)
		0–0.105226 (vs. *Hirst.* sp.)		- (vs. *Hirst.* sp.)
*L. h*	0	0–0.114634 (vs. *E. s*)	0	- (vs. *E. s*)
		0–0.113034 (vs. *Hg. n*)		0–0.304940 (vs. *Hg. n*)
		0–0.128336 (vs. *H. m*)		0–0.211069 (vs. *H. m*)
		0–0.105137 (vs. *M. g*)		0–0.320741 (vs. *M. g*)
		0–0.118999 (vs. *Hirst.* sp.)		- (vs. *Hirst.* sp.)
*E. s*	0–0.002626	0–0.022748 (vs. *Hg. n*)	-	- (vs. *Hg. n*)
		0–0.169238 (vs. *H. m*)		- (vs. *H. m*)
		0–0.024086 (vs. *M. g*)		- (vs. *M. g*)
		0–0.043391 (vs. *Hirst.* sp.)		- (vs. *Hirst.* sp.)
*Hg. n*	0–0.002632	0–0.162658 (vs. *H. m*)	0	0–0.337478 (vs. *H. m*)
		0–0.026818 (vs. *M. g*)		0–0.344651 (vs. *M. g*)
		0–0.046214 (vs. *Hirst.* sp.)		- (vs. *Hirst.* sp.)
*H. m*	0	0–0.165662 (vs. *M. g*)	0.088065	0–0.332020 (vs. *M. g*)
		0–0.179227 (vs. *Hirst.* sp.)		- (vs. *Hirst.* sp.)
*M. g*	0	0–0.040487 (vs. *Hirst.* sp.)	0	- (vs. *Hirst.* sp.)
*Hirst.* sp.	0	-	-	-

Abbreviations: *L. a—Laelaps agilis*, *L. j—Laelaps jettmari*, *L. h—Laelaps hilaris*, *E. s—Eulaelaps stabularis*, *Hg. n—Haemogamasus nidi*, *H. m—Hyperlaelaps microti*, *M. g—Myonyssus gigas*, *Hirst.* sp.—*Hirstionyssus* sp., - no data available. ^a^ Mean distances are shown between species.

**Table 4:** Hosts and GenBank nucleotide accession numbers of the COI gene sequences of Laelapidae mites.

Taxonomic Status of Species	Host Species	Country	Length (bp)	GenBank Accession No.	AT%	No of Representative Samples
Genus *Laelaps*						
*L. agilis* (1 hap.)	*A. fla*	Slovakia	699	MZ315167	74.1	1
	*A. fla*	Slovakia	695	MZ315168	74.4	3
	*A. fla*	Lithuania	650	ON754956	73.8	4
	*C. gla*	Lithuania	651	ON754955	73.7	2
	*A. syl*	Czech Republic	685	MZ315172	74.5	1
	*C. gla*	Czech Republic	651	OP199248	73.7	1
*L. agilis* (2 hap.)	*A. syl*	Czech Republic	684	MZ315169	74.3	1
	*A. syl*	Czech Republic	688	MZ315171	74.2	1
*L. agilis* (3 hap.)	*A. syl*	Czech Republic	684	MZ315170	73.5	1
*L. agilis* (4 hap.)	*A. agr*	Lithuania	582	MZ048460	74.6	1
	*A. agr*	Lithuania	582	MZ048461	74.6	1
	*C. gla*	Lithuania	582	MZ048462	74.6	2
	*A. fla*	Lithuania	646	ON754957	74.0	7
*L. agilis* (5 hap.)	*C. gla*	Lithuania	582	MZ048463	74.2	1
*L. agilis* (6 hap.)	*M. oec*	Lithuania	582	MZ048464	74.4	1
	*A. fla*	Lithuania	649	ON754958	73.7	1
*L. agilis* (7 hap.)	*C. gla*	Lithuania	647	ON754963	73.7	2
	*A. fla*	Lithuania	650	ON754962	73.7	2
	*A. fla*	Lithuania	650	ON754961	73.7	5
	*M. min*	Lithuania	649	ON754960	73.7	1
	*C. gla*	Lithuania	652	ON754959	73.5	3
*L. agilis* (8 hap.)	*A. fla*	Lithuania	650	ON754964	73.8	1
*L. agilis* (9 hap.)	*A. fla*	Norway	650	ON754965	73.1	1
*L. jettmari* (1 hap.)	*A. agr*	Lithuania	582	MZ048465	73.9	1
*L. jettmari* (2 hap.)	*A. agr*	Lithuania	582	MZ048466	73.7	1
*L. jettmari* (3 hap.)	*A. agr*	Lithuania	657	OP199246	72.0	1
	*A. agr*	Lithuania	645	OP199245	72.2	1
*L. hilaris*	*A. agr*	Lithuania	582	MZ048455	72.0	1
	*M. arv*	Lithuania	582	MZ048456	72.0	1
	*M. arv*	Lithuania	582	MZ048457	72.0	1
Genus *Haemogamasus*						
*Hg. nidi*	*A. fla*	Lithuania	582	MZ049956	64.9	1
	*C. gla*	Lithuania	582	MZ049957	64.9	1
Genus *Hyperlaelaps*						
*H. microti* (1 hap.)	*M. arv*	Lithuania	582	MZ048467	73.7	1
*H. microti* (2 hap.)	*M. oec*	Lithuania	582	MZ048468	72.3	1
Genus *Myonyssus*						
*M. gigas*	*C. gla*	Lithuania	582	MZ048469	70.1	1
	*C. gla*	Lithuania	582	MZ048470	70.1	1

Abbreviations: *A. agr—Apodemus agrarius*, *A. fla—Apodemus flavicollis*, *A. syl—Apodemus sylvaticus*, *M. arv—Microtus arvalis*, *M. oec—Microtus oeconomus*, *C. gla—Clethrionomys glareolus*.

**Table 5:** Comparison of the COI gene sequences of *L. agilis* mites in this study.

Nucleotide Position			No of Representative Samples						1	1	1	1	1	2	2	2	3	3	3	3	4	4	5	5	5	5
				1	3	4	7	9	3	5	6	7	7	0	1	8	0	2	7	9	5	8	1	1	2	4
Samples				5	3	8	5	0	8	6	2	1	2	1	6	8	9	1	2	6	0	3	0	6	2	9
1 HAP	MZ315167 *A. fla* SK		1	A	T	A	T	T	A	G	A	G	G	A	A	G	A	A	A	T	T	G	A	G	A	A
	MZ315168 *A. fla* SK		3	.	.	.	.	.	.	.	.	.	.	.	.	.	.	.	.	.	.	.	.	.	.	.
	ON754956 *A. fla* LT		4	.	.	.	.	.	.	.	.	.	.	.	.	.	.	.	.	.	.	.	.	.	.	.
	ON754955 *C. gla* LT		2	.	.	.	.	.	.	.	.	.	.	.	.	.	.	.	.	.	.	.	.	.	.	.
	MZ315172 *A. syl* CZ		1	.	.	.	.	.	.	.	.	.	.	.	.	.	.	.	.	.	.	.	.	.	.	.
	OP199248 *C. gla* CZ		1	.	.	.	.	.	.	.	.	.	.	.	.	.	.	.	.	.	.	.	.	.	.	.
2 HAP	MZ315171 *A. syl* CZ		1	.	.	.	.	C	.	.	.	.	.	.	.	.	.	.	.	.	.	.	.	.	.	.
	MZ315169 *A. syl* CZ		1	.	.	.	.	C	.	.	.	.	.	.	.	.	.	.	.	.	.	.	.	.	.	.
3 HAP	MZ315170 *A. syl* CZ		1	T	.	G	.	.	G	A	G	.	.	.	G	A	T	G	T	A	A	A	G	A	G	.
4 HAP	MZ048462 *C. gla* LT		2	.	.	.	.	.	.	.	.	A	.	.	.	.	.	.	.	.	.	.	.	.	.	G
	MZ048461 *A. agr* LT		1	.	.	.	.	.	.	.	.	A	.	.	.	.	.	.	.	.	.	.	.	.	.	G
	MZ048460 *A. agr* LT		1	.	.	.	.	.	.	.	.	A	.	.	.	.	.	.	.	.	.	.	.	.	.	G
	ON754957 *A. fla* LT		7	.	.	.	.	.	.	.	.	A	.	.	.	.	.	.	.	.	.	.	.	.	.	G
5 HAP	MZ048463 *C. gla* LT		1	.	.	.	C	.	.	.	.	.	.	.	.	.	.	.	.	.	.	.	.	.	.	G
6 HAP	MZ048464 *M. oec* LT		1	.	.	.	.	.	.	.	.	.	.	.	.	.	.	.	.	.	.	.	.	.	.	G
	ON754958 *A. fla* LT		1	.	.	.	.	.	.	.	.	.	.	.	.	.	.	.	.	.	.	.	.	.	.	G
7 HAP	ON754963 *C. gla* LT		2	.	.	.	C	.	.	.	.	.	.	.	.	.	.	.	.	.	.	.	.	.	.	.
	ON754962 *A. fla* LT		2	.	.	.	C	.	.	.	.	.	.	.	.	.	.	.	.	.	.	.	.	.	.	.
	ON754961 *A. fla* LT		5	.	.	.	C	.	.	.	.	.	.	.	.	.	.	.	.	.	.	.	.	.	.	.
	ON754960 *M. min* LT		1	.	.	.	C	.	.	.	.	.	.	.	.	.	.	.	.	.	.	.	.	.	.	.
	ON754959 *C. gla* LT		3	.	.	.	C	.	.	.	.	.	.	.	.	.	.	.	.	.	.	.	.	.	.	.
8 HAP	ON754964 *A. fla* LT		1	.	.	.	C	.	.	.	.	.	A	.	.	.	.	.	.	.	.	.	.	.	.	.
9 HAP	ON754965 *A. fla* NO		1	T	C	.	.	.	G	A	G	.	.	G	G	A	T	G	T	A	A	A	G	A	G	.

Abbreviations: *L. a—Laelaps agilis*, *A. agr—Apodemus agrarius*, *A. fla—Apodemus flavicollis*, *A. syl—Apodemus sylvaticus*, *C. gla—Clethrionomys glareolus*, *M. oec—Microtus oeconomus*; *M. min—Micromys minutus*, LT—Lithuania, SK—Slovakia, CZ—Czech Republic, NO—Norway.

## Data Availability

The data presented in this study are available within the article.
